# The Diabetes-Linked Transcription Factor PAX4: From Gene to Functional Consequences

**DOI:** 10.3390/genes8030101

**Published:** 2017-03-09

**Authors:** Petra I. Lorenzo, Francisco Juárez-Vicente, Nadia Cobo-Vuilleumier, Mario García-Domínguez, Benoit R. Gauthier

**Affiliations:** 1 Pancreatic Islet Development and Regeneration Unit, Department of Cell Regeneration and Advanced Therapies, CABIMER (Junta de Andalucía-CSIC-Universidad de Sevilla-Universidad Pablo de Olavide), Calle Américo Vespucio, 24, 41092 Sevilla, Spain; nadia.cobo@cabimer.es; 2 Cell differentiation Lab, Department of Cell Signaling and Dynamics, CABIMER (Junta de Andalucía-CSIC-Universidad de Sevilla-Universidad Pablo de Olavide), Calle Américo Vespucio, 24, 41092 Sevilla, Spain; francisco.juarez@cabimer.es (F.J.-V.); mario.garcia@cabimer.es (M.G.-D.)

**Keywords:** PAX4, transcription regulation, SUMOylation, diabetes mellitus, β-cell adaptation, regenerative therapy

## Abstract

Paired box 4 (PAX4) is a key factor in the generation of insulin producing β-cells during embryonic development. In adult islets, PAX4 expression is sequestered to a subset of β-cells that are prone to proliferation and more resistant to stress-induced apoptosis. The importance of this transcription factor for adequate pancreatic islets functionality has been manifested by the association of mutations in *PAX4* with the development of diabetes, independently of its etiology. Overexpression of this factor in adult islets stimulates β-cell proliferation and increases their resistance to apoptosis. Additionally, in an experimental model of autoimmune diabetes, a novel immunomodulatory function for this factor has been suggested. Altogether these data pinpoint at PAX4 as an important target for novel regenerative therapies for diabetes treatment, aiming at the preservation of the remaining β-cells in parallel to the stimulation of their proliferation to replenish the β-cell mass lost during the progression of the disease. However, the adequate development of such therapies requires the knowledge of the molecular mechanisms controlling the expression of PAX4 as well as the downstream effectors that could account for PAX4 action.

## 1. Introduction

Diabetes Mellitus (DM) is a metabolic disease characterized by hyperglycemia due to defects in insulin secretion by pancreatic β-cells, insulin action on target tissues or a combination of both [1]. Due to the dramatic rise in DM incidence in the last decades, this disease has become a leading health problem worldwide; according to the International Diabetes Federation (www.idf.org), the number of people affected by DM is expected to increment from current estimates of 415 million to 642 million by the year 2040. This escalation in DM cases is mainly due to the increase of Type 2 Diabetes Mellitus (T2DM) that nowadays accounts for as much as 90% of all diabetes cases. The main underlying reason is the increased prevalence of obesity in our society. Nevertheless, although overweight is a major factor contributing to T2DM, only 20%–40% of obese individuals develop T2DM [2]. This fact, together with the existence of family history of DM in type 2 diabetic patients indicates that T2DM has a strong genetic component. Interestingly, the majority of the identified T2DM susceptibility genes are related to β-cell function, indicating that alterations in the performance of β-cells which are unable to compensate for the increased insulin demand in obese individuals, is the main triggering factor of the disease. However, these susceptibility loci only explain 10%–20% of T2DM heritability [3,4,5]. One of the possible reasons is the polygenic nature of T2DM, caused by the interplay between different genetic variants and environmental factors. Therefore, single polymorphisms with minor impact would be associated with T2DM only when several of them are combined and genetic and environmental interactions are taken into account [6,7]. This fact could explain, at least in part, the apparent ethnic specificity of some of T2DM susceptibility genes, such as with the Paired box 4 (*PAX4*). Two single nucleotide polymorphisms (SNPs), rs6467136 and rs10229583, in intergenic regions close to *PAX4* have been associated with T2DM by genome-wide association studies (GWAS) in Asian populations [8,9]. Moreover, mutations in *PAX4* have been associated, not only with the development of T2DM and with one of the Maturity-Onset Diabetes of the Young (MODY) subtypes, MODY9 in East Asian families [10,11], but also with ketosis prone diabetes in individuals of West African origin and with Type 1 Diabetes Mellitus (T1DM) in some European populations. Therefore, *PAX4* is one of the few genes whose polymorphisms/mutations have been associated with several forms of DM [10]. Altogether, this data pinpoints at *PAX4* as a key DM susceptibility gene, marking it as a valuable target for the development of new therapies for DM treatment independently of the disease etiology. Harnessing the genetic, epigenetic and posttranslational mechanisms regulating PAX4 levels/activity is mandatory for the adequate development of novel approaches for DM treatment.

## 2. PAX4 in Islet Physiology: Key Player in β-Cell Generation, Survival and Proliferation

PAX4 belongs to the Pax gene family, a group of evolutionary conserved transcription factors involved in embryonic organogenesis as well as in cell plasticity in the adult [12,13,14,15,16]. PAX4 is mainly expressed in the endocrine pancreas where it plays an essential role in the generation of insulin producing β-cells during embryonic development and later on, during adulthood, is a β-cell master regulator in adaptation processes [10,17,18,19].

### 2.1. PAX4 Essential Role for β-Cell Generation during Embryogenesis

During pancreas development PAX4 is initially expressed in all endocrine progenitors [20,21], being thereafter implicated in the commitment of β/δ progenitors and further development of β-cells [22,23]. The lack of this gene in mouse models leads to the near absence of β- and δ-cells, coupled to an increase in the number of α-cells, rendering the animals severely hyperglycemic leading to neonatal death [22,23,24]. Further evidence on PAX4 triggering β-cell commitment arises from transgenic mouse models where induction of PAX4 expression in early pancreatic epithelium, or in endocrine committed precursor cells induces the formation of insulin producing β-cells at the expense of all other islet cell phenotypes [25]. In agreement with this, ectopic expression of PAX4 potentiates the in vitro generation of insulin^+^ β-like cells [26,27,28,29,30,31,32].

### 2.2. PAX4 Implications in Adult Islet Plasticity

In adult pancreatic islets, PAX4 is implicated in β-cell plasticity as evidenced by both in vitro and in vivo studies. Treatments of β-cell lines and rodent islets with mitogens (such as activin A or betacellulin) or with high glucose increase both *PAX4* expression and β-cell proliferation [33,34]. Moreover, ectopic expression of mouse PAX4 in human or rat adult islets enhances β-cell proliferation [33]. Strikingly, human PAX4, in contrast to its mouse counterpart, does not induce proliferation in isolated islets [34]. Besides this pro-proliferative role, PAX4 expression has also been linked to increased β-cell survival. Induction of endogenous *PAX4* levels or ectopic expression of this factor have been associated with increased expression of anti-apoptotic members of the intrinsic apoptotic pathway, correlating with improved survival of β-cells and higher resistance to cytokine-induced apoptosis [33,35,36,37]. Silencing PAX4 in insulinoma cell lines decreases the expression of anti-apoptotic factors concomitantly with the increase in spontaneous apoptosis as well as with higher sensitivity to cytokine-induced cell death [38]. The pro-proliferative and anti-apoptotic role of PAX4 has been validated in vivo in a mouse model that conditionally over-express PAX4 specifically in β-cells. Over-expression of PAX4 in vivo protects β-cells from apoptosis induced by streptozotocin (STZ) and in a mouse model of experimental autoimmune diabetes (RIPB7.1 mice) [37,39]. Remarkably, in the latter model, PAX4 overexpression decreases islet immune cell infiltration (insulitis), suggesting a novel immunomodulatory function of PAX4 [39].

Interestingly, long term ectopic expression of PAX4 in vivo increases proliferation of PDX1^+^ β-cells that express low to undetectable levels of insulin [37]. This increase in β-cell proliferation results in blunted glucose stimulated insulin secretion (GSIS) correlating with altered islet morphology, effects that are reversed upon inhibition of PAX4 expression. These data suggest a PAX4-mediated dedifferentiation of mature β-cells to allow their proliferation. In agreement with this, in islet β-cell tumors induced by the loss of Menin1 (Men1), lesions become progressively INS^−^ concomitantly with the increase in activin B and PAX4 levels [40]. Likely activin B mediated activation of *Pax4* expression contributes to the dedifferentiation of β-cells observed during progression of *Men1*^-/-^ tumors [40]. The correlation of sustained PAX4 expression with dedifferentiation of β-cells may seem contradictory with the fact that PAX4 is expressed in β-cells of adult islets. Possibly, a threshold level of PAX4 is required to trigger PAX4 dedifferentiation potential. Moreover, the well-known heterogeneity among islet β-cells is also evidenced in PAX4 expression. A recent report from our group has shown that, in adult mouse islets, PAX4 expression is only detected in a subpopulation of β-cells that are prone to proliferation and more resistant to apoptosis. Furthermore, the relative abundance of this subpopulation of PAX4^+^β-cells correlates with the proliferative capacity of the islets [21], leading us to speculate that this subpopulation of PAX4^+^β-cells is responsible for β-cell plasticity in response to external stimuli in vivo.

Besides the important role of PAX4 on β-cells, experimental ectopic PAX4 expression in α-cells in vivo induces their transdifferentiation into functional β-like cells independently of mice age [25,41]. This PAX4-mediated transdifferentiation of α-cells has also been proven in vitro after adenoviral overexpression of PAX4 in the α-TC cell line [42]. Moreover, intraperitoneal injection of these transdifferentiated β-like cells into hyperglycemic mice results in transient improvement of the glycemic levels [42]. The possibility of transdifferentiating α-cells into functional β-like cells by the overexpression of a single transcription factor reinforces the concept of PAX4 as a β cell master regulator. However, in agreement with the required decrease of PAX4 levels to allow final maturation of β-cells, transdifferentiated monohormonal insulin^+^ cells express lower levels of PAX4 as compared to bihormonal intermediate glucagon^+^/insulin^+^ cells [42].

Altogether, these data indicate that PAX4 plays a crucial role in β-cell commitment during development, in the maintenance of more responsive adult β-cell able to adapt to environmental challenges as well as in the transdifferentiation of α-cells into functional β-cells. This pinpoints at PAX4 as an important target for the development of novel therapies aiming at the regeneration of the β-cell mass lost during the progression of DM. Thus, understanding the genetics of PAX4 regulation as well as identification of its downstream target genes is a priority.

## 3. PAX4 Molecular Structure and Mechanism of Action

**Figure 1:**
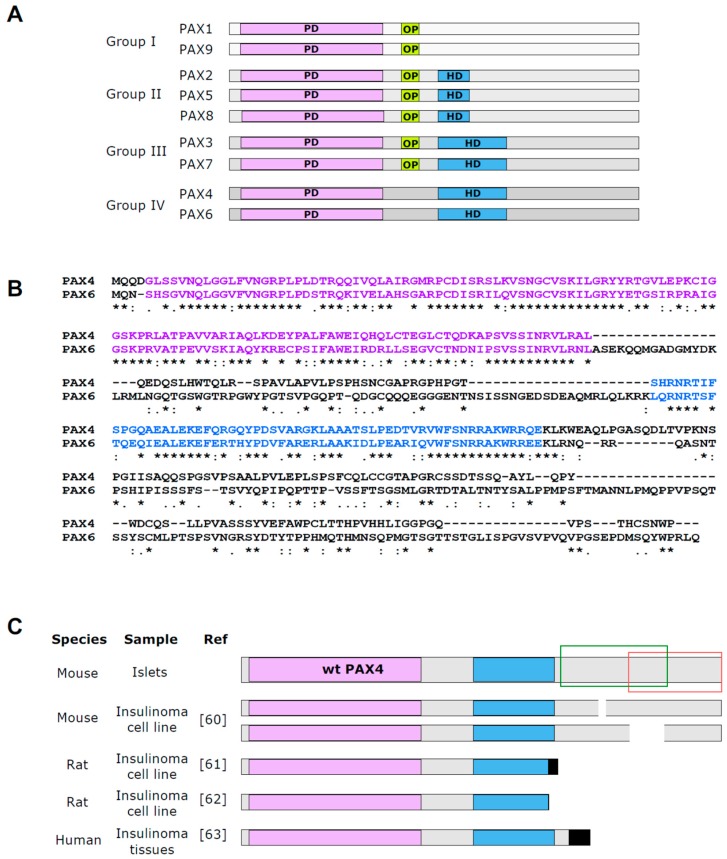
PAX4 DNA binding domains share high homology with PAX6. (**A**) Schematic representation of Pax family members and their division into four groups based on their structural domains: paired domain (PD), octapetide (OP) and homeodomain (HD). (**B**) Alignment of mouse PAX4 (UniProtKB P32115) and mouse PAX6 (UniProtKB P63015) protein sequences using Clustal Omega Analysis Tool Web Services from the EMBL-EBI. Conservation code: {*} fully conserved residue {:} conservation between groups of strongly similar properties and {.} conservation between groups of weakly similar properties. (**C**) Schematic representation of full length PAX4 protein identified in islets and the truncated variants found in insulinoma samples. Color code: PD is indicated in purple, HD in blue and OP in yellow. Black indicates mutation caused new sequence unrelated to PAX4. Green box indicates the region where the transactivation domain (TAD) is located and red box the region including the repressor domain.

PAX4, as well as other members of the Pax family, are sequence specific transcription factors that exert their action through binding to defined cis-acting sequences located in both promoters and enhancers of target genes [43]. In mammals, Pax family is comprised of nine members (PAX1 to PAX9) divided into four groups based on the organization of three conserved structural motifs: the highly conserved 127–128 amino acid DNA binding paired domain (PD) common to all family members, a complete or truncated DNA interacting homeodomain (HD), and the octapeptide (OP) motif located between the PD and the HD (Figure 1A). PAX4, together with PAX6, constitute group IV of the Pax family, characterized by the presence of the PD and a complete HD, but lacking the OP [44]. PAX4 and PAX6 are highly homologous in the PD and HD (70% and 65%, respectively), with no obvious homology outside these domains [44] (Figure 1B).

The presence of two DNA binding domains (DBDs) in these transcription factors confers a high complexity in their DNA sequence recognition. The N-terminal PD, comprising the main DBD, is on its own a complex bipartite structure composed of two helix-turn-helix (HTH) motifs separated by a flexible conserved polypeptide chain. The N-terminal subdomain, termed PAI, is a highly conserved region that confers DNA sequence recognition. The C-terminal subdomain, the RED subdomain, is more divergent and, despite presenting lower affinity for DNA in some PAX proteins, can modify the binding specificity of the PD [45,46,47,48,49]. The flexible linker between these two HTH motifs also establishes contact with DNA [47]. The second DBD, the HD, can also bind independently and with high affinity to specific DNA sequences; nevertheless, a cooperative action of the PD and the HD on DNA recognition has been documented [50], and functional interdependence between these two domains regulates PAX proteins binding to their target gene regulatory elements [48,51,52,53]. This double DBD with possibility of independent vs. cooperative action confers extraordinary flexibility of sequence recognition to each PAX protein hindering the correct prediction of recognition sites by sequence analysis of the putative target genes. Nonetheless, analysis of PAX4 binding sites using either the isolated PAX4 PD [54] or a truncated form of PAX4 that contains both DBDs [44], determined that PAX4 PD binding site has a seven-nucleotide core similar to the consensus motifs identified for other PAX proteins. Nevertheless, it is important to note that in these two studies some differences in the identified consensus sequence were observed. While in the study using only the PD the recognized sequence is (G/T)T(C/G)A(T/A)GC, the identified consensus motif for the PD when both DBDs are present in the same protein is ANNN(C/T)CACCC. One can speculate that these differences can be due to the presence or not of the HD that can modify the binding characteristics of the PD. The binding site for PAX4 HD itself contains the consensus TAAT motif characteristic for HD containing factors [44]. Nevertheless, due to limitations of the approach used, the existence of other important sites for specificity of the binding cannot be excluded.

Because of the high conservation between PAX4 and PAX6 DBDs, the consensus binding site for PAX4 is similar, but not identical, to the consensus for PAX6. This similarity can explain the capacity of PAX4 PD as well as the full length protein to bind to PAX6 sites, however with less affinity than PAX6, allowing competition of both factors for the same targets [44,54,55]. However, the lower affinity of PAX4 for these binding sites together with its lower expression in pancreatic β-cells as compared to PAX6 also suggest that these two factors have different and specific target genes. The functional importance of PAX4 DBDs is clearly established by the fact that the majority of PAX4 mutations associated with diabetes are located within these domains [10,11]. One of the best characterized diabetes-linked mutations of PAX4 is R121W (homolog to R129W in mouse). This single amino acid substitution in the PD of PAX4 alters the DNA binding capacity of the factor [33,56], leading to an impaired protection of β-cells against apoptosis [37].

In addition to its DNA binding properties, the PD can also engage in protein–protein interactions that modulate the transcriptional activity of the factor. Both PAX3 and PAX6 interact with members of the Sox family, resulting in synergistic transcription activation of specific target genes [57,58]. The PD of PAX6 is needed for this interaction [57]. Moreover, PAX4 and PAX6 also interact via the PD of PAX6. The region of PAX4 involved in this binding has not yet been characterized [55]. Based on the sequence homology between the PDs of PAX4 and PAX6, it can be anticipated that PAX4 PD is also involved in protein–protein interactions; however, putative partners of PAX4 remain uncharacterized. In addition, the PD can also act as a protein transduction domain (PTD). PAX4 intact PD, as well as PAX6 and PAX5 PDs allow protein transduction into living cells. Incubation of different cell lines as well as isolated rat islets with recombinant PAX4 protein results in the entrance of functional PAX4 protein into cells [35]. The PTDs are normally highly basic peptides, however PAX4 PD has a low content of basic amino acids. This fact together with the requirement of an intact PD suggests that the conserved 3D structure of PAX proteins PD is determinant for efficient transduction.

The C-terminal region of PAX proteins is the more divergent part among the different members of the family. This region is rich in Proline/Serine/Threonine and contains a transactivation domain (TAD). Precise localization of these TADs requires further characterization [49,59]. In some members, as in PAX6, the TAD activity seems to be independent of cell type [43]. However, the activity of the PAX4 TAD (located in a region between amino acids 232 to 314 of mouse PAX4) (Figure 1C) is cell-type dependent, and often associated with E1A-like activity, a marker of undifferentiated cell status [54]. One unique characteristic of PAX4 is the presence of a negative regulatory domain (within the amino acids 274 to 349 of mouse PAX4) juxtaposed to the TAD (Figure 1C). This domain confers a transcription repressor function to PAX4 that can hinder the activity of other TADs in fused proteins [54]. This repressor domain is active regardless of cell type, suggesting that it interacts with a ubiquitous co-repressor or exerts its action directly through interaction with the transcription machinery [54]. Interestingly, insulinomas express truncated variants of PAX4 characterized by modified C-terminal domains with functional PD and HD [60,61,62,63] (Figure 1C). In vitro functional analysis of such a variant revealed that this truncated protein binds DNA as efficiently as the wild type (WT) protein, but activates rather than repress gene transcription [63]. Although further studies are required to elucidate the physiological role of PAX4 in insulinomas, it is tempting to speculate that this transcription activator role of truncated PAX4 may be one of the underlying reasons of the uncontrolled cell proliferation. In addition to this repressor domain in the C-terminal end of PAX4 protein, the existence of a second repressor domain located between amino acids 2 and 230 has also been suggested, however the precise location of this domain has not yet been characterized [54,62].

## 4. PAX4 Mechanism of Action: Downstream Regulated Genes

Since in islet β-cells PAX4 and PAX6 are co-expressed and PAX4 can bind to the same binding sites as PAX6, the interaction between these two factors will determine the regulatory outcome of different target genes [10]. The functional relevance of this interaction was analyzed in reporter assays using PAX6 consensus sites upstream of a minimal promoter. Increasing concentration of PAX4 competed with PAX6 for binding, resulting in decreased reporter expression [44,54]. This decrease is the result of two different processes, the blocking of PAX6-dependent activation and the inhibitory action of PAX4 repressor domain [54].

In an attempt to elucidate the molecular mechanisms underlying PAX4 action on β-cell phenotype the effect of PAX4 on key islet cells genes has been studied. Analysis of the effect of PAX4 onto different islet hormones expression has revealed that PAX4 inhibits the expression of glucagon, ghrelin and insulin. Ectopic expression of PAX4 in α-cell lines results in inhibition of glucagon gene expression [55,64]. This action is mediated, at least in part, by specific competition with PAX6 for the binding to G1 and/or G3 elements of the glucagon gene promoter [55]. Interestingly, human PAX4 has less affinity than mouse PAX4 for this glucagon G3 element [34]. This DNA binding differences between the two homologs could justify the lack of proliferative capacity of human PAX4 in β-cells [34]. PAX4 also binds to the ghrelin gene promoter (at a site located in a 1.3 kb fragment upstream of the transcription start site (TSS)) directly repressing transcription [65]. Remarkably, PAX6 has no effect on ghrelin expression, excluding competition between PAX4 and PAX6 for the same site as the underlying mechanism. Interestingly the lack of PAX4 during embryonic development despite not affecting single hormone ghrelin^+^ ε-cell population does induce the ectopic activation of ghrelin in glucagon^+^ α-cells [65]. PAX4 also binds to a region in the human insulin gene promoter (between −229 and−258) that contains a C2 and E2 site, mediating transcriptional repression [62]. Interestingly, an independent study analyzing the effect of PAX4 on the C2 element revealed that while in α-cell lines PAX4 causes a sharp decrease in transcriptional activation, in β-cell lines it has little effect, indicating some degree of cell-type specificity [44]. PAX4 dependent decrease of insulin expression was also observed after in vivo overexpression of PAX4 in β-cells [37]. Interestingly, the ectopic expression of mutant PAX4R129W that has impaired DNA binding [33,56], also decreases insulin expression. This suggests the involvement of a PAX4 DNA-binding independent mechanism in this genetic regulation. There are no reports regarding PAX4 role onto somatostatin expression, however PAX6 stimulated somatostatin expression through binding to an upstream enhancer [66]. We could also expect a PAX4–PAX6 interaction, resulting in repression of somatostatin gene transcription by PAX4. Therefore, PAX4 is a key repressor of islet hormone genes. The inhibition of glucagon or ghrelin by PAX4 will ensure the formation of single-hormone expressing cells. This is consistent with the exclusion of PAX4 from α-cells to ensure glucagon expression. This exclusion process is mediated through ARX (aristaless related homeobox) that triggers α-cell formation. ARX and PAX4 inhibit each other expression through binding to specific DNA sites. PAX4 binds to a conserved enhancer region in *Arx*, located 14.2 kb downstream of the translation stop site, inhibiting *Arx* transcription. On the other hand, ARX binds to *Pax4* in a conserved pancreatic enhancer region inhibiting *Pax4* expression. This mutual inhibition ensures proper endocrine fate [24].

PAX4-dependent inhibition of insulin promoter paradoxically suggests that high PAX4 expression maintains β-cells in an immature status. In agreement with this, PAX4 can also inhibit the expression of several mature β-cell markers. PAX4 binds to and represses the promoter of islet amyloid polypeptide (IAPP or amylin), which is co-secreted with insulin [62]. MAFA, an important factor during the acquisition of glucose responsiveness in neonatal β-cells [67], is inhibited by ectopic expression of PAX4 in β-cells in vivo [37]. This action is mediated, at least in part, through the conserved R3 region of *MafA* located 8 kb upstream of the TSS [37,68]. Interestingly, PAX4 mutant variant PAX4R129W has a similar inhibitory effect, suggesting a DNA-binding-independent action of PAX4. Since PAX6 also binds to the R3 region [69] activating *MafA* transcription, PAX4–PAX6 direct protein–protein interaction might be, at least in part, mediating this negative action of PAX4 [37]. The expression of the glucose transporter 2 (GLUT2, also termed SLC2A2) is also downregulated by PAX4 overexpression in vivo [37]. Similar to *MafA* and insulin regulation, mutant PAX4R129W can also inhibit the expression of *Glut2*. It is tempting to speculate that in PAX4 dependent regulation of β-cell maturation a DNA-binding independent action of PAX4 has a prominent role, however further studies to identify PAX4 interaction partners are required to validate this hypothesis.

The existence in adult islets of a subpopulation of insulin-expressing β-cells that specifically express PAX4 [21], suggests that PAX4 expression above a specific threshold is required for inhibition of these mature β-cell markers. In support of this hypothesis, only after long term ectopic expression of PAX4 in β-cells in vivo emerges a proliferative PDX1^+^/insulin^−^ cell population, indicative of the dedifferentiation of the β-cells [37]. Thus, PAX4 expression induces dedifferentiation of β-cells allowing their proliferation. One of the downstream targets that can account for PAX4 dependent activation of β-cell proliferation is c-Myc. Ectopic expression of mouse PAX4 in human or rat adult islets in culture induces c-Myc expression correlating with β-cell proliferation [33,35]. Moreover, long-term in vivo ectopic expression of PAX4 increases *c-Myc* and *Cdk4* mRNA levels, correlating with the increase in proliferation of PDX1^+^/insulin^−^ β-cells [37]. Remarkably, transcriptome analysis of islet cells after PAX4 overexpression in vivo revealed the downregulation of cyclin-dependent kinase inhibitor 2A, which strongly inhibits *Cdk4*, [39]. Further analysis or these microarray data revealed a functional enrichment in cell cycle pathway after PAX4 overexpression. In silico analysis of this pathway indicated that overexpression of PAX4 increases the expression of both cell cycle activators and cell cycle inhibitors, suggesting that PAX4 defines a proliferation permissive β-cell subpopulation primed to expansion only under conditions that alleviate cell cycle breaks [21].

PAX4 is also a survival factor for β-cells. Several members of the intrinsic apoptotic pathway are modulated after increased PAX4 expression. The expression of the anti-apoptotic factor *Bcl-xL* is increased after overexpression of PAX4 in an insulinoma cell line as well as in isolated rat islets [34,38]. In addition, the treatment of MIN6 mouse insulinoma cell line with recombinant PAX4 protein protects these cells from TNF-α induced apoptosis, with a concomitant increase in *Bcl-xL* expression [35]. In contrast, silencing of PAX4 expression in INS-1E insulinoma cell line decreases the expression levels of *Bcl-xL* correlating with increased spontaneous apoptosis and higher sensitivity to cytokines-induced apoptosis [38]. Additionally, an association between increased PAX4 expression and increased BCL-2 levels, another anti-apoptotic factor of the intrinsic pathway, has been reported in neonatal rat islets after treatment with ciliary neurotrophic factor (CNTF) that promotes β-cell survival [36]. Ectopic expression of PAX4 in mouse islet β-cells also correlates with an increase in *Bcl-2* expression and abrogated cytochrome C release after cytokines treatment, demonstrating the important role of PAX4 on the intrinsic apoptotic pathway. Remarkably, PAX4R129W overexpression does not increase *Bcl-2* or protect mouse islet β-cells from cytokines-induced apoptosis [37]. Supporting this, in vivo overexpression of PAX4 renders mouse β-cells more resistant to STZ induced apoptosis, while PAX4R129W confers only a partial protection [37]. The impairment of DNA binding of the mutant variant and its failure in apoptosis protection suggest that PAX4 dependent regulation of the intrinsic apoptotic pathway requires PAX4 binding to its specific DNA sites. Reinforcing this hypothesis, the increase in *Bcl-xL* expression in rat islets is more pronounced after ectopic expression of mouse PAX4 as compared to human PAX4 that has a less efficient binding to the G3 element of the glucagon promoter [34]. In addition to *Bcl-2* and *Bcl-xL*, other genes involved in cell survival are upregulated by PAX4, but not by PAX4R129W.

Transcriptome profiling on islets overexpressing PAX4 in β-cells in vivo revealed a functional enrichment of protein processing in endoplasmic reticulum pathway, not enriched in PAX4R129W overexpressing islets [39]. PAX4 role in the endoplasmic reticulum (ER) response was demonstrated after treatment of isolated islets with thapsigargin, an inhibitor of ER Ca^2+^-ATPases, which induces ER-stress dependent apoptosis. PAX4 overexpressing islets showed preservation of ER integrity and decreased apoptosis after thapsigargin treatment. This protection was lost when PAX4R129W mutant was overexpressed [39]. Silencing of endogenous PAX4 in MIN6 sensitized cells to thapsigargin-induced apoptosis, clearly demonstrating the importance of PAX4 in maintaining ER homeostasis [39]. In this context, PAX4 increased expression of calreticulin (CALR), a Ca2^+^ binding protein involved in ER homeostasis whereas PAX4R129W decreased its transcript levels. PAX4 was also recently shown to protect β-cells against apoptosis in an experimental model of autoimmune diabetes correlating with a significant decrease in islet immune cells infiltration (insulitis), suggesting the involvement of this transcription factor in immune modulation. Although the mechanism remains to be defined, a potential target implicated in this process that was increased by PAX4 but not PAX4R129W is Galectin 9 (LGALS9) [39]. LGALS9 is known to have potent immunomodulatory functions, capable of reducing insulitis and hyperglycemia in Non Obese Diabetic (NOD) mice [70,71], as well as prolonging islet grafts survival [72]. A PAX4-dependent increase in *Lgals9* expression might be one of the mechanisms implicated in PAX4 immunomodulatory action. In PAX4-mediated regulation of these pro-survival genes, the PD of the transcription factor has a major role, since the PAX4R129W mutant fails to activate their expression. Therefore, we can hypothesize that PAX4 dependent binding to DNA is required for this regulation. Whether it is a direct regulation or intermediate factors are required remains to be determined.

## 5. PAX4 Regulation

In adult mouse islets, PAX4 expression is restricted to a subpopulation of β-cells that are prone to proliferation and more resistant to apoptosis. The relative abundance of this subpopulation of PAX4^+^β-cells increases under physiological situations that require an increase in the proliferation of β-cells such as pregnancy [21] indicating a stringent regulation of this factor even within a single cell phenotype.

### 5.1. Epigenetic Regulation of PAX4

**Figure 2:**
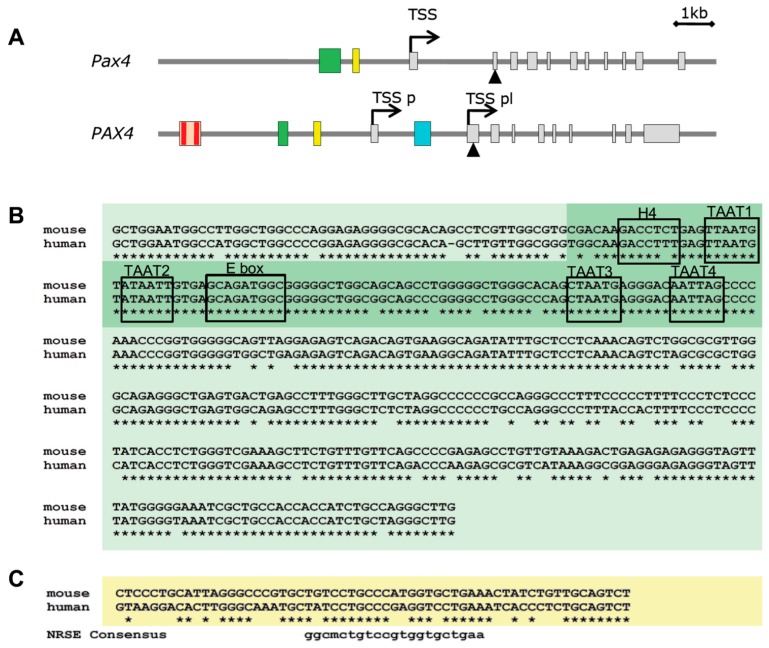
Mouse *Pax4* gene and regulatory upstream region. (**A**) Schematic structure of mouse *Pax4* (top) and human *PAX4* (bottom) loci according to UCSC Genome Browser annotations (http://genome.ucsc.edu/). Exons are depicted as grey squares. Transcription start site (TSS) is indicated by an arrow. In human *PAX4* two different TSS have been defined: TSS pl: placental TSS and TSS p: pancreatic TSS. Triangle represents first codon. Green box represents the promoter region that directs pancreatic expression of PAX4. Yellow box indicates the localization of the neuron-restrictive silencer element (NRSE). Red box represents the localization of the PAX4 binding sites in human promoter. Cyan box represents the demethylated region in hematologic malignancies. (**B**) Alignment of human and mouse 0.4 kb promoter region that directs pancreatic expression of PAX4. In dark green is indicated the 0.1 kb region identified in human. Boxes indicate the location of the potential transcription factors binding sites. (**C**) Alignment of human and mouse NRSE.

Several epigenetic modifications have been shown to control *Pax4* expression. Treatment of rat islets with a DNA-methyltransferase inhibitor, 5′AZA, stimulates endogenous *Pax4* expression and consequently expression of its downstream target *Bcl-xL*, suggesting that demethylation of *Pax4* promoter is an epigenetic modification controlling *Pax4* expression [34]. Moreover, demethylation of the human *PAX4* promoter region located −1347 to −1103 bp from translation initiation site (Figure 2A) has been linked to aberrant expression of *PAX4* in lymph nodes of patients with diffuse large B cell lymphoma (DLBCL) as well as in several hematological cell lines [73]. Of note, hematological organs do not express *PAX4* under physiological conditions. In agreement with this, treatment of lymphocyte cell lines devoid of *PAX4* expression with 5′AZA robustly induces expression of *PAX4*. In addition, treatment of pancreatic explants with MG1568, an inhibitor of class IIa histone deacetylases (HDACs) enhanced the expression of *Pax4* [74]. This activation was transient reaching a peak of expression between days 5 and 9 in culture reverting to basal levels by day 11. Noteworthy, by day 11, insulin and *MafA* expression were significantly increased in MG1568-treated explants [74]. These findings suggest that *Pax4* activation promotes dedifferentiation and expansion but that repression is mandatory to achieve β-cell maturation with expression of insulin.

### 5.2. Genetic Regulation of PAX4

Studies aiming at the characterization of *PAX4* functional promoter identified a highly conserved (88% sequence homology between mouse and human) 0.4 kb fragment located 2 kb upstream of the mouse *Pax4* TSS (Figure 2) that is sufficient for driving *Pax4* expression in pancreas in vivo[75,76]. This region is hypomethylated in β- and non-β-cells of adult mouse islets [21]. In an independent study, a 0.1 kb region located 2 kb upstream of human *PAX4* TSS was found to be both necessary and sufficient to drive pancreas specific expression of *PAX4* [77]. This human pancreas specific promoter element is located within the highly conserved 0.4 kb fragment identified in mice (Figure 2B), highlighting the importance of this region for PAX4 regulation in pancreas. Hepatocyte nuclear factor 4 alpha (HNF4α), HNF1 homeobox A (HNF1α), PDX1, and neuronal differentiation 1 (NEUROD1) / neurogenin 3 (NEUROG3) were shown to interact within this region [77] (Figure 2B). Interestingly, among these five putative regulators of *PAX4*, four of them are established Maturity-Onset Diabetes of the Young (MODY) genes: HNF4α-MODY1, HNF1α-MODY3, PDX1-MODY4 and NEUROD1-MODY6. Further functional studies in non-β cells showed that a combination of these factors, HNF4α, HNF1α, PDX1 and NEUROD1, together with PAN1 increased expression of a reporter construct harboring this promoter region while alone they had no impact. Substituting NEUROD1 by NEUROG3 further increased activation indicating that these factors are likely involved in the regulation of *PAX4* during pancreas organogenesis [77]. Further analysis of these sites in a β-cell line revealed that mutation in either the HNF1α or NEUROD1/NEUROG3 binding site caused a strong decrease in the promoter activity, while mutation of HNF4α site had a minor effect [78]. Expression of NEUROG3 and HNF1α together, but not individually, induced endogenous *PAX4* transcription in a non-pancreatic cell line. These data indicate that *PAX4* activation requires the co-expression of NEUROG3 and HNF1α [78]. In agreement with this, ectopic expression of NEUROG3 alone in pancreatic ductal cells that express endogenous HNF1α, was able to induce endogenous *PAX4* expression [78,79]. NEUROD1, a downstream target of NEUROG3 that binds to the same site as NEUROG3 in the *PAX4* promoter also induced expression of endogenous *PAX4* in pancreatic ductal cells, but not in HeLa cells. However, noteworthy is the fact that after *NEUROG3* ectopic expression in ductal cells PAX4 is increased prior to NEUROD1 stimulation [79]. In addition to these binding sites, the 5′ end fragment of the human *PAX4* promoter (−4958 to −2153) contains two additional binding sites for PAX4 (Figure 2) mediating a negative feedback loop on its own expression [77]. Accordingly, reporter assays using the full-length 4958 bp promoter (containing the two putative binding sites), showed that co-expression with PAX4 repressed the activity of the promoter, and that this effect was more prominent in α-TC cells. Removal of the 5′ region containing these putative binding sites resulted in a milder repression by PAX4.

Interestingly, 0.5 kb downstream of the pancreas specific promoter element a 21 bp cis-regulatory motif for a neuron-restrictive silencer factor (NRSF, also termed REST) was identified (Figure 2A). This *Pax4* NRSE (neuron-restrictive silencer element) is conserved throughout evolution (Figure 2B) and confers NRSF-dependent transcriptional repression of *Pax4* [80]. Due to the fact that REST is not expressed in neuronal cells nor in β-cells [81,82] this mechanism of repression is not expected to be active in β-cells and will probably be involved in regulation of the specific pattern of expression of PAX4. Moreover, high mobility group 20A (HMG20A, also known as IBRAF), a chromatin remodeling factor implicated in the relief of transcriptional repression induced by the LSD1-CoREST complex, is expressed in pancreatic islets, and mutations in this gene have been associated with T2DM [83]. Further studies are required to establish the link between PAX4 expression and HMG20A regulatory function.

Different treatments known to stimulate β-cell proliferation have been associated with activation of *Pax4* expression. Treatment of rodent islets with mitogens, such as activin A (belonging to TGF-β family) or betacellulin (belonging to EGF family) increases β-cell proliferation concomitantly with the induction of *Pax4* expression [33]. Inhibition of the PI3-kinase pathway by betacellulin abrogates induction of *Pax4* expression and subsequent β-cell proliferation, revealing the implication of this pathway in the activation of *Pax4* expression. Moreover, the treatment of pancreatic explants with betacellulin promotes β-cell fate at the expense of α-cell destiny [84]. In contrast, TGF-β1, another member of the TGF-β family, does not have a significant effect on Pax4 mRNA levels, or on islet cell proliferation [13,33]. In addition, *Pax4* expression in human islets is also increased under high glucose concentration in vitro. Most likely insulin released in response to high glucose is the main stimulator of *Pax4* transcription, as blocking insulin secretion inhibits the increase in *Pax4* mRNA, while insulin treatment mimics the effect of high glucose [18]. Interestingly further increase in glucose concentration (33 mM) caused *Pax4* repression, probably due to high levels of IL-1β [18]. It is tempting to speculate that during the initial stages of T2DM the increase in blood glucose may have a stimulatory action on *Pax4* expression, favoring the proliferation of β-cells detected in these patients. Further increase in glucose levels or maybe longer time in the presence of high blood glucose, could be mediating opposite effect by increasing IL-1β and therefore inhibiting *Pax4* expression contributing to β-cell death observed during later stages of the disease. Strengthening the possible involvement of high glucose levels on PAX4 expression, endogenous *PAX4* mRNA levels are increased in T2DM donors with body mass index (BMI) between 22 and 26. Higher BMI donors have lower expression levels of *PAX4* indicating that long term exposure and lipotoxicity suppresses *PAX4* expression [18].

### 5.3. Posttranslational Regulation of PAX4

In addition to genetic and epigenetic regulation, posttranslational modifications (PTMs) are likely involved in modulating PAX4 activity, yet little is known. One such PTMs is SUMOylation that regulates transcription factors function by fine-tuning the activity of the factor by modulating protein stability, cellular localization, DNA binding and interaction with partners [85,86,87,88,89,90]. As such SUMOylation was shown to regulate the activity of key β-cell proteins such as MAFA, PDX1 and Glukokinase [91]. Of particular interest, SUMO-4 was identified as a candidate gene implicated in Type 1 diabetes susceptibility [92]. Furthermore, increased SUMOylation as well as overexpression of SUMO1 protects INS-1 cells against IL-1β induced apoptosis. In contrast, ectopic expression of the deSUMOylating enzyme SENP1 sensitizes this cell line to apoptosis induced by IL-1β [93]. These effects are reminiscent of either PAX4 overexpression protecting against cytokine-induced apoptosis or its repression sensitizing to apoptosis. Interestingly, SUMOylation regulates the transcriptional activity of PAX6, by modulating the binding activity (Yan, et al. 2010). The SUMOylation site within PAX6 is conserved in PAX4 suggesting the potential SUMOylation of PAX4.

**Figure 3:**
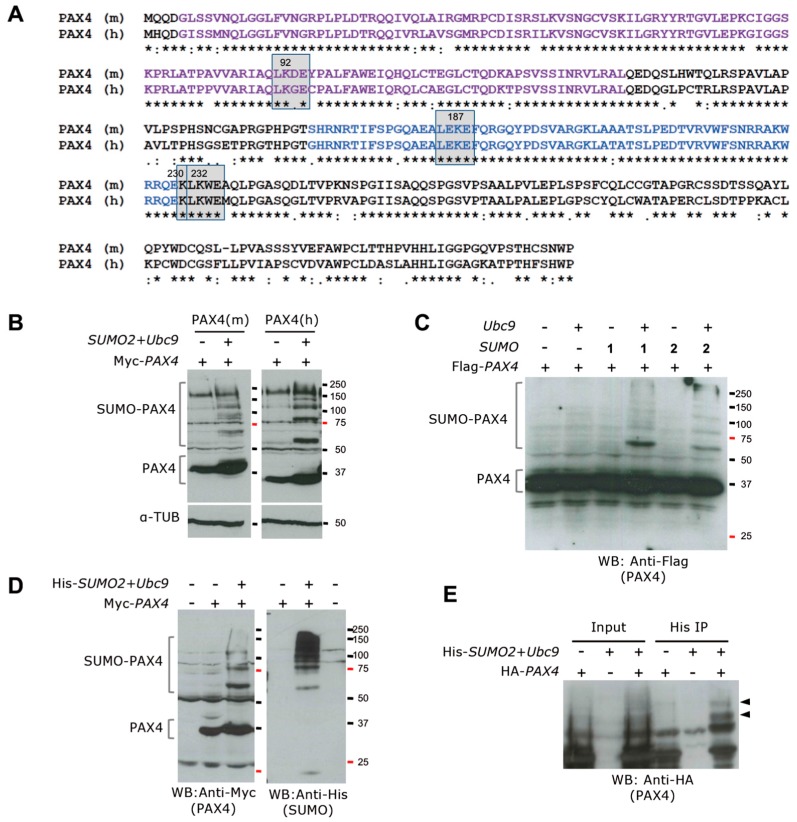
Mouse and human PAX4 proteins are SUMOylated. (**A**) Alignment of mouse PAX4 (PAX4 (m) UniProtKB P32115) and human PAX4 (PAX4 (h) UniProtKB O43316) protein sequences using Clustal Omega. PD is indicated in purple and HD in blue. The predicted SUMOylation sites are indicated by grey squares. (**B**) Western blot of cellular extracts from 293T cells transfected with Myc-tagged mouse *Pax4* (left panels) or human *PAX4* (right panels) alone or in combination with *SUMO2* and *Ubc9*. Co-transfection of *PAX4* with *SUMO2* and *Ubc9* results in the detection of SUMOylated PAX4. α-Tubulin was used as loading control (lower panels). Interestingly, human PAX4 SUMOylation is stronger when compared with mouse PAX4. In both cases, the co-expression of *SUMO2* and *Ubc9* seems to increase the stability of PAX4 protein, as indicated by the stronger band for unSUMOylated PAX4. (**C**) Western blot analysis of 293T cells transfected with Flag-tagged *PAX4* alone or in combination with *Ubc9*, *SUMO1* or *SUMO2*, shows human PAX4 SUMOylation by both SUMO1 and SUMO2 only when Ubc9 is co-expressed. (**D**) Analysis of extracts of 293T cells untransfected or transfected with Myc-tagged *PAX4* alone or in combination with His-Tagged *SUMO2* and *Ubc9*, using anti-Myc (left panel) or anti-His (right panel) antibodies, revealed the specificity of the SUMOylated bands. The lower band ~20 kD in the anti-His blot corresponds to free SUMO. (**E**) Pull down using His-Trap matrix of extracts from 293T cells transfected with Ha-tagged *PAX4* alone or in combination with His-tagged *SUMO2* and *Ubc9* showing the specific pull down of SUMOylated PAX4, indicated by arrows.

In silico analysis of PAX4 revealed the presence of several additional putative SUMOylation sites conserved in human and mouse PAX4 proteins (Figure 3). Two SUMOylation consensus sites (ψKXE where ψ is a hydrophobic residue and X is any amino acid) with the SUMO acceptor Lys at position 92 and 232 were predicted. Moreover, adjacent to the 232-site a second Lys that could also be SUMOylated was identified in both proteins. These analyses prompt us to investigate whether human and mouse PAX4 were SUMOylation targets. Our studies show that both human and mouse PAX4 proteins are SUMOylated by SUMO1 and SUMO2 in the presence of the SUMO conjugating enzyme Ubc9 and without the requirement of any of the protein inhibitor of activated STAT (PIAS) SUMO E3 ligases (Figure 3). This opens a new regulatory level of PAX4 activity. Mutational analysis of the putative SUMO acceptor Lys will determine the main site for SUMOylation for PAX4 and reveal whether this PTM can modify the transcription regulation potential of PAX4.

## 6. Conclusions

**Figure 4:**
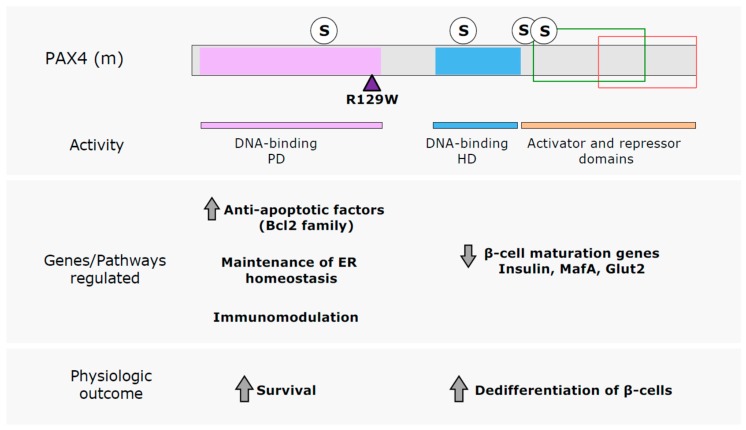
PAX4 mechanism of action. The differences observed in the regulation mediated by PAX4 WT and the diabetes linked mutant PAX4R129W has revealed the existence of, at least, two different mechanisms of action for PAX4. The main DBD of PAX4, the PD, is required for the upregulation of genes and pathways implicated in the increased survival of β-cells, while the dedifferentiation of β-cells seems to be independent of this DBD. R129W mutation and predicted SUMOylation sites (S) are indicated in the schematic representation of mouse PAX4 (PAX4 (m)) protein. PD is indicated in purple, HD in blue. Green box indicates the region where the TAD is located and red box the region including the repressor domain.

PAX4 is a key factor regulating β-cell survival and proliferation in adult islet β-cells, therefore an interesting target for the development of novel therapies for DM treatment aimed at sustaining a functional β-cell mass. The activation of endogenous *Pax4* expression is an adaptive response intrinsic of the β-cells as revealed by the described correlation between the abundance of the PAX4^+^ β-cell population and the proliferative capacity of the islets [21]. Moreover, the increase in *Pax4* expression in mice treated with STZ [94], together with the higher resistance of PAX4^+^ β-cells to STZ induced apoptosis [21] indicates that *Pax4* stimulation is also a mechanism of protection inherent of the β-cells. Therefore, stimulating agents that activate this β-cell response could be used for enhancing proliferation and resistance to apoptosis in β-cells. However, long term high expression of PAX4 can induce dedifferentiation of the β-cells causing islets dysfunction. Therefore, wide knowledge on the genetics of PAX4 mechanism of action is needed, to be able to potentiate PAX4 protection against cell death without causing the detrimental dedifferentiation of the β-cells. In this regard, the comparative functional analysis of PAX4 and a mutant variant associated with DM, R129W, has revealed the existence of, at least, two different mechanism of action for this transcription factor [37,39] (Figure 4). On one the hand, PAX4 acts as a survival factor for β-cells, regulating different anti-apoptotic members of the Bcl2 family as well as preserving ER homeostasis, through a mechanism that requires an intact PD, since PAX4R129W (mutation located in the PD) does not regulate these genes/pathways. As a result of R129W mutant PAX4 has impaired DNA binding, therefore this pro-survival action of PAX4 is mediated through binding to consensus response elements in PAX4 target genes. On the other hand, high PAX4 levels decrease the expression of mature β-cell markers indicating dedifferentiation of the β-cells, and remarkably PAX4R129W has the same effect as the WT protein. Thus, this mechanism of action of PAX4 seems to be independent of its binding to the conventional response elements. Protein–protein interaction mechanisms, as described for PAX4–PAX6, could be mediating this effect. Nevertheless, since the HD of PAX4R129W remains intact, binding to DNA sites specific for this second DBD could also be involved. However, further studies are required to validate this hypothesis. It is tempting to speculate that understanding the molecular and genetic mechanisms underlying these different actions of PAX4 could convey in a development of a better response, by increasing the protective actions of PAX4 without inducing β-cell dedifferentiation. This is highly relevant in the case of diabetic patients harboring mutations in PAX4 PD. These mutations, similar to R129W, may hamper the pro-survival action of PAX4 while retaining its β-cell dedifferentiation capacity. Identification of PAX4 downstream targets that account for PAX4 anti-apoptotic actions may lead to the identification of new druggable targets that will result in a more appropriate therapeutic intervention for these individuals.

The finding that PAX4 is SUMOylated opens a new level of regulation of its activity. In silico analysis of PAX4 indicates that at least four Lys conserved between human and mouse PAX4 could be SUMOylated. SUMOylation of transcription factors has been frequently associated with a decrease in their transcriptional activity, due to the sequestering of the factors in nuclear bodies or inactivation of their TADs. However, in the case of PAX6, K91 SUMOylation in p32 PAX6 (isoform of PAX6 that lacks the PD) activates the DNA binding of the HD, thus stimulating the transcriptional activity of this isoform (Yan, et al. 2010). This site is conserved in PAX4 (K187 in mouse PAX4) and could thus also be involved in modulating PAX4 HD binding activity. Similarly, the SUMO site located in PAX4 PD (K92 in mPax4) could be involved in the modulation of the binding to specific sites for this DBD. Interestingly the third and fourth putative SUMOylation sites (K230 and K232 in mouse PAX4) are located in the C-terminal region of PAX4, which contains a TAD and a repressor domain. SUMOylation close to these types of domains have been shown to decrease their activity [89], and could be a mechanism of regulation of PAX4 activity. However, mutational analyses to identify the SUMO acceptor Lys to characterize the functional consequence of this PTM are necessary.

The possibility of transdifferentiating α-cells into functional β-cells by ectopic expression of PAX4 [25,41,42] opens new possibilities towards the development of novel gene therapeutic approaches to treat DM. This mechanism will not only provide an alternative to increase β-cell mass, but also maintaining an α to β cell ratio, important for adequate glucose homeostasis. Moreover, the proof of concept in mouse models that intra-bile ductal injection of adenoviruses expressing human PAX4 provided therapeutic benefits [41,42] points at the use of *PAX4* in gene therapy.

The capacity of functional PAX4 protein to enter into living cells opens a new venue of PAX4 itself as a therapeutic tool. Different studies have focused on the use of PTDs and cell penetrating peptides (CCPs) for the delivery of therapeutic proteins in the treatment of diseases [95]. Therefore, PAX4 innate capacity to transduce into living cells maintaining its functionality is advantageous. Understanding PAX4 mechanisms of action as well as the functional effect of SUMOylation on this transcription factor could encourage the generation of genetically modified PAX4 proteins with reduced dedifferentiation potential and increased protective actions that can be used as therapeutic proteins. However, due to the unspecific cell penetration of PAX4, adequate delivery platforms need to be developed. Additionally, further analyses of the penetration efficiency of PAX4 are necessary to determine the applicability of this approach. Nevertheless, it is an appealing strategy to modify the expression/action of PAX4 without genetic manipulation.

**Figure 5:**
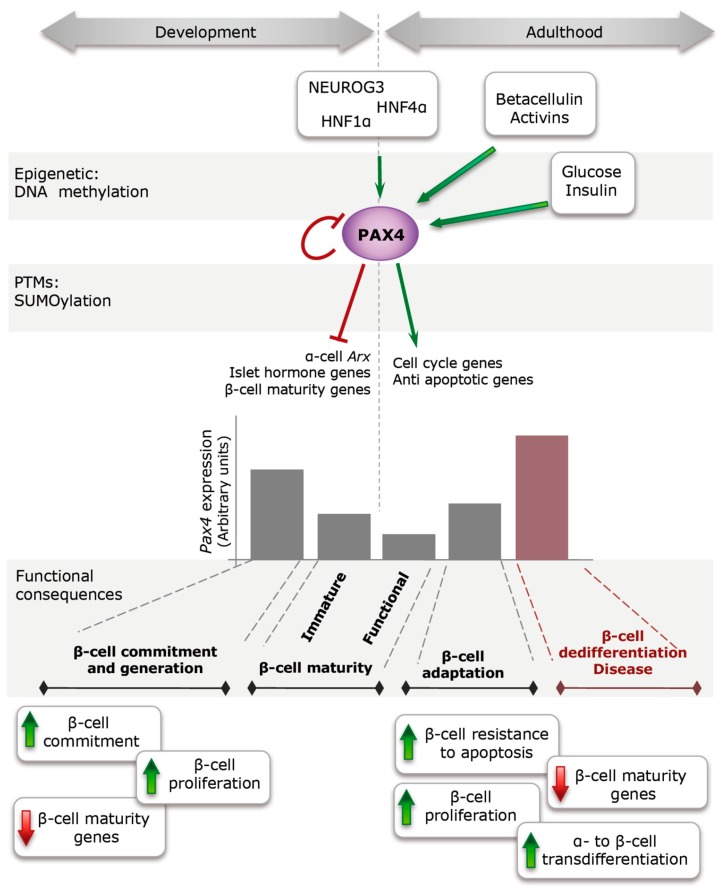
PAX4 regulation and action during development and adulthood. During embryonic development, NEUROG3, HNF1α and probably HNF4α activate PAX4 expression in progenitor cells inducing their commitment towards β-cells through inhibition of other islet cell master factors (ARX and islet hormone genes). Later on during development, PAX4 expression is tune down to allow full expression of β-cell markers (MAFA, GLUT2, insulin) conferring full functionality to β-cells during early post-natal life. During adulthood, increases in the insulin demand induce *Pax4* expression to confer a more plastic behavior of the β-cells (increase in apoptosis resistance and prone to proliferation) that allows the adaptation of the islets to the new metabolic demands. However, if *Pax4* stimulation is maintained for long term, it can cause the dedifferentiation of the β-cells (decrease in mature β-cell markers), with subsequent inability to regulate glucose homeostasis.

In conclusion, lessons learnt from PAX4 genetic regulations and physiologic effects (Figure 5) sustain the possible future use of PAX4 in regenerative therapies. In addition, stimulation of the expression/activity of this transcription factor could also be beneficial in islets transplantation for DM treatment. In vitro treatment of isolated islets to stimulate PAX4 expression/activity prior to their transplant could result in increased survival of the grafts and thus improved outcome for the patient.

## References

[1] Association A.D. (2014). Diagnosis and classification of diabetes mellitus. Diabetes Care.

[2] Nguyen N.T., Nguyen X.M., Lane J., Wang P. (2011). Relationship between obesity and diabetes in a US adult population: Findings from the National Health and Nutrition Examination Survey, 1999–2006. Obes. Surg..

[3] Imamura M., Maeda S. (2011). Genetics of type 2 diabetes: The GWAS era and future perspectives [review]. Endocr. J..

[4] Boitard C., Accili D., Ahren B., Cerasi E., Seino S., Thorens B. (2012). The hyperstimulated beta-cell: Prelude to diabetes?. Diabetes Obes. Metab..

[5] Groop L., Pociot F. (2014). Genetics of diabetes—Are we missing the genes or the disease?. Mol. Cell. Endocrinol..

[6] Chatterjee N., Wheeler B., Sampson J., Hartge P., Chanock S.J., Park J.H. (2013). Projecting the performance of risk prediction based on polygenic analyses of genome-wide association studies. Nat. Genet..

[7] Zuk O., Hechter E., Sunyaev S.R., Lander E.S. (2012). The mystery of missing heritability: Genetic interactions create phantom heritability. Proc. Natl. Acad. Sci. USA.

[8] Cho Y.S., Chen C.H., Hu C., Long J., Ong R.T., Sim X., Takeuchi F., Wu Y., Go M.J., Yamauchi T. (2012). Meta-analysis of genome-wide association studies identifies eight new loci for type 2 diabetes in east asians. Nat. Genet..

[9] Ma R.C., Hu C., Tam C.H., Zhang R., Kwan P., Leung T.F., Thomas G.N., Go M.J., Hara K., Sim X. (2013). Genome-wide association study in a chinese population identifies a susceptibility locus for type 2 diabetes at 7q32 near pax4. Diabetologia.

[10] Martin-Montalvo A., Lorenzo P.I., Lopez-Noriega L., Gauthier B.R. (2016). Targeting pancreatic expressed PAX genes for the treatment of diabetes mellitus and pancreatic neuroendocrine tumors. Expert Opin. Ther. Targets.

[11] Sujjitjoon J., Kooptiwut S., Chongjaroen N., Tangjittipokin W., Plengvidhya N., Yenchitsomanus P.T. (2016). Aberrant mRNA splicing of paired box 4 (PAX4) IVS7–1G>A mutation causing maturity-onset diabetes of the young, type 9. Acta Diabetol..

[12] Robson E.J., He S.J., Eccles M.R. (2006). A PANorama of PAX genes in cancer and development. Nat. Rev. Cancer.

[13] Lang D., Powell S.K., Plummer R.S., Young K.P., Ruggeri B.A. (2007). PAX genes: Roles in development, pathophysiology, and cancer. Biochem. Pharmacol..

[14] Blake J.A., Thomas M., Thompson J.A., White R., Ziman M. (2008). Perplexing Pax: From puzzle to paradigm. Dev. Dyn..

[15] Wang Q., Fang W.H., Krupinski J., Kumar S., Slevin M., Kumar P. (2008). Pax genes in embryogenesis and oncogenesis. J. Cell. Mol. Med..

[16] Blake J.A., Ziman M.R. (2014). Pax genes: Regulators of lineage specification and progenitor cell maintenance. Development.

[17] Sosa-Pineda B. (2004). The gene Pax4 is an essential regulator of pancreatic beta-cell development. Mol. Cells.

[18] Brun T., Gauthier B.R. (2008). A focus on the role of Pax4 in mature pancreatic islet β-cell expansion and survival in health and disease. J. Mol. Endocrinol..

[19] Napolitano T., Avolio F., Courtney M., Vieira A., Druelle N., Ben-Othman N., Hadzic B., Navarro S., Collombat P. (2015). Pax4 acts as a key player in pancreas development and plasticity. Semin. Cell Dev. Biol..

[20] Greenwood A.L., Li S., Jones K., Melton D.A. (2007). Notch signaling reveals developmental plasticity of Pax4(+) pancreatic endocrine progenitors and shunts them to a duct fate. Mech. Dev..

[21] Lorenzo P.I., Fuente-Martin E., Brun T., Cobo-Vuilleumier N., Jimenez-Moreno C.M., Irene G.H.G., Lopez Noriega L., Mellado-Gil J.M., Martin-Montalvo A., Soria B. (2015). Pax4 defines an expandable beta-cell subpopulation in the adult pancreatic islet. Sci. Rep..

[22] Sosa-Pineda B., Chowdhury K., Torres M., Oliver G., Gruss P. (1997). The Pax4 gene is essential for differentiation of insulin-producing beta cells in the mammalian pancreas. Nature.

[23] Wang J., Elghazi L., Parker S.E., Kizilocak H., Asano M., Sussel L., Sosa-Pineda B. (2004). The concerted activities of Pax4 and Nkx2.2 are essential to initiate pancreatic beta-cell differentiation. Dev. Biol..

[24] Collombat P., Hecksher-Sorensen J., Broccoli V., Krull J., Ponte I., Mundiger T., Smith J., Gruss P., Serup P., Mansouri A. (2005). The simultaneous loss of Arx and Pax4 genes promotes a somatostatin-producing cell fate specification at the expense of the alpha- and beta-cell lineages in the mouse endocrine pancreas. Development.

[25] Collombat P., Xu X., Ravassard P., Sosa-Pineda B., Dussaud S., Billestrup N., Madsen O.D., Serup P., Heimberg H., Mansouri A. (2009). The ectopic expression of Pax4 in the mouse pancreas converts progenitor cells into alpha and subsequently beta cells. Cell.

[26] Blyszczuk P., Czyz J., Kania G., Wagner M., Roll U., St-Onge L., Wobus A.M. (2003). Expression of Pax4 in embryonic stem cells promotes differentiation of nestin-positive progenitor and insulin-producing cells. Proc. Natl. Acad. Sci. USA.

[27] Lin H.T., Kao C.L., Lee K.H., Chang Y.L., Chiou S.H., Tsai F.T., Tsai T.H., Sheu D.C., Ho L.L., Ku H.H. (2007). Enhancement of insulin-producing cell differentiation from embryonic stem cells using pax4-nucleofection method. World J. Gastroenterol..

[28] Liew C.G., Shah N.N., Briston S.J., Shepherd R.M., Khoo C.P., Dunne M.J., Moore H.D., Cosgrove K.E., Andrews P.W. (2008). PAX4 enhances beta-cell differentiation of human embryonic stem cells. PLoS ONE.

[29] Lima M.J., Docherty H.M., Chen Y., Docherty K. (2012). Efficient differentiation of AR42J cells towards insulin-producing cells using pancreatic transcription factors in combination with growth factors. Mol. Cell. Endocrinol..

[30] Berneman-Zeitouni D., Molakandov K., Elgart M., Mor E., Fornoni A., Dominguez M.R., Kerr-Conte J., Ott M., Meivar-Levy I., Ferber S. (2014). The temporal and hierarchical control of transcription factors-induced liver to pancreas transdifferentiation. PLoS ONE.

[31] Gage B.K., Baker R.K., Kieffer T.J. (2014). Overexpression of PAX4 reduces glucagon expression in differentiating hESCs. Islets.

[32] Soria B., Gauthier B.R., Martin F., Tejedo J.R., Bedoya F.J., Rojas A., Hmadcha A. (2015). Using stem cells to produce insulin. Expert Opin. Biol. Ther..

[33] Brun T., Franklin I., St-Onge L., Biason-Lauber A., Schoenle E., Wollheim C.B., Gauthier B.R. (2004). The diabetes-linked transcription factor Pax4 promotes beta-cell proliferation and survival in rat and human islets. J. Cell Biol..

[34] Brun T., He K.H., Lupi R., Boehm B., Wojtusciszyn A., Sauter N., Donath M., Marchetti P., Maedler K., Gauthier B.R. (2008). The diabetes-linked transcription factor Pax4 is expressed in human pancreatic islets and is activated by mitogens and GLP-1. Hum. Mol. Genet..

[35] Lu J., Li G., Lan M.S., Zhang S., Fan W., Wang H., Lu D. (2007). Pax4 paired domain mediates direct protein transduction into mammalian cells. Endocrinology.

[36] Rezende L.F., Stoppiglia L.F., Souza K.L., Negro A., Langone F., Boschero A.C. (2007). Ciliary neurotrophic factor promotes survival of neonatal rat islets via the BCL-2 anti-apoptotic pathway. J. Endocrinol..

[37] He K.H.H., Lorenzo P.I., Brun T., Jimenez Moreno C.M., Aeberhard D., Ortega J.V., Cornu M., Thorel F., Gjinovci A., Thorens B. (2011). In Vivo conditional Pax4 overexpression in mature islet {beta}-cells prevents stress-induced hyperglycemia in mice. Diabetes.

[38] Brun T., Duhamel D.L., He K.H.H., Wollheim C.B., Gauthier B.R. (2007). The transcription factor Pax4 acts as a survival gene in the insulinoma INS1E cells. Oncogene.

[39] Mellado-Gil J.M., Jimenez-Moreno C.M., Martin-Montalvo A., Alvarez-Mercado A.I., Fuente-Martin E., Cobo-Vuilleumier N., Lorenzo P.I., Bru-Tari E., de Gracia Herrera-Gomez I., Lopez-Noriega L. (2016). PAX4 preserves endoplasmic reticulum integrity preventing beta cell degeneration in a mouse model of type 1 diabetes mellitus. Diabetologia.

[40] Ripoche D., Charbord J., Hennino A., Teinturier R., Bonnavion R., Jaafar R., Goehrig D., Cordier-Bussat M., Ritvos O., Zhang C.X. (2016). ActivinB is induced in insulinoma to promote tumor plasticity through a beta-cell-induced dedifferentiation. Mol. Cell. Biol..

[41] Al-Hasani K., Pfeifer A., Courtney M., Ben-Othman N., Gjernes E., Vieira A., Druelle N., Avolio F., Ravassard P., Leuckx G. (2013). Adult duct-lining cells can reprogram into beta-like cells able to counter repeated cycles of toxin-induced diabetes. Dev. Cell.

[42] Zhang Y., Fava G.E., Wang H., Mauvais-Jarvis F., Fonseca V.A., Wu H. (2016). Pax4 gene transfer induces α-to-β cell phenotypic conversion and confers therapeutic benefits for diabetes treatment. Mol. Ther..

[43] Czerny T., Busslinger M. (1995). DNA-binding and transactivation properties of Pax-6: Three amino acids in the paired domain are responsible for the different sequence recognition of Pax-6 and BSAP (Pax-5). Mol. Cell. Biol..

[44] Smith S.B., Ee H.C., Conners J.R., German M.S. (1999). Paired-homeodomain transcription factor PAX4 acts as a transcriptional repressor in early pancreatic development. Mol. Cell. Biol..

[45] Czerny T., Schaffner G., Busslinger M. (1993). DNA sequence recognition by Pax proteins: Bipartite structure of the paired domain and its binding site. Genes Dev..

[46] Epstein J.A., Glaser T., Cai J., Jepeal L., Walton D.S., Maas R.L. (1994). Two independent and interactive DNA-binding subdomains of the Pax6 paired domain are regulated by alternative splicing. Genes Dev..

[47] Xu H.E., Rould M.A., Xu W., Epstein J.A., Maas R.L., Pabo C.O. (1999). Crystal structure of the human Pax6 paired domain-DNA complex reveals specific roles for the linker region and carboxy-terminal subdomain in DNA binding. Genes Dev..

[48] Apuzzo S., Abdelhakim A., Fortin A.S., Gros P. (2004). Cross-talk between the paired domain and the homeodomain of Pax3: DNA binding by each domain causes a structural change in the other domain, supporting interdependence for DNA binding. J. Biol. Chem..

[49] Mayran A., Pelletier A., Drouin J. (2015). Pax factors in transcription and epigenetic remodelling. Semin. Cell Dev. Biol..

[50] Jun S., Desplan C. (1996). Cooperative interactions between paired domain and homeodomain. Development.

[51] Singh S., Stellrecht C.M., Tang H.K., Saunders G.F. (2000). Modulation of PAX6 homeodomain function by the paired domain. J. Biol. Chem..

[52] Mishra R., Gorlov I.P., Chao L.Y., Singh S., Saunders G.F. (2002). PAX6, paired domain influences sequence recognition by the homeodomain. J. Biol. Chem..

[53] Yan Q., Gong L., Deng M., Zhang L., Sun S., Liu J., Ma H., Yuan D., Chen P.C., Hu X. (2010). Sumoylation activates the transcriptional activity of Pax-6, an important transcription factor for eye and brain development. Proc. Natl. Acad. Sci. USA.

[54] Fujitani Y., Kajimoto Y., Yasuda T., Matsuoka T.A., Kaneto H., Umayahara Y., Fujita N., Watada H., Miyazaki J.I., Yamasaki Y. (1999). Identification of a portable repression domain and an E1A-responsive activation domain in Pax4: A possible role of Pax4 as a transcriptional repressor in the pancreas. Mol. Cell. Biol..

[55] Ritz-Laser B., Estreicher A., Gauthier B.R., Mamin A., Edlund H., Philippe J. (2002). The pancreatic beta-cell-specific transcription factor Pax-4 inhibits glucagon gene expression through Pax-6. Diabetologia.

[56] Shimajiri Y., Sanke T., Furuta H., Hanabusa T., Nakagawa T., Fujitani Y., Kajimoto Y., Takasu N., Nanjo K. (2001). A missense mutation of Pax4 gene (R121W) is associated with type 2 diabetes in Japanese. Diabetes.

[57] Kamachi Y., Uchikawa M., Tanouchi A., Sekido R., Kondoh H. (2001). Pax6 and SOX2 form a co-DNA-binding partner complex that regulates initiation of lens development. Genes Dev..

[58] Lang D., Epstein J.A. (2003). Sox10 and Pax3 physically interact to mediate activation of a conserved c-RET enhancer. Hum. Mol. Genet..

[59] Glaser T., Jepeal L., Edwards J.G., Young S.R., Favor J., Maas R.L. (1994). PAX6 gene dosage effect in a family with congenital cataracts, aniridia, anophthalmia and central nervous system defects. Nat. Genet..

[60] Inoue H., Nomiyama J., Nakai K., Matsutani A., Tanizawa Y., Oka Y. (1998). Isolation of full-length cDNA of mouse PAX4 gene and identification of its human homologue. Biochem. Biophys. Res. Commun..

[61] Tokuyama Y., Yagui K., Sakurai K., Hashimoto N., Saito Y., Kanatsuka A. (1998). Molecular cloning of rat Pax4: Identification of four isoforms in rat insulinoma cells. Biochem. Biophys. Res. Commun..

[62] Campbell S.C., Cragg H., Elrick L.J., Macfarlane W.M., Shennan K.I., Docherty K. (1999). Inhibitory effect of Pax4 on the human insulin and islet amyloid polypeptide (IAPP) promoters. FEBS Lett..

[63] Miyamoto T., Kakizawa T., Ichikawa K., Nishio S., Kajikawa S., Hashizume K. (2001). Expression of dominant negative form of PAX4 in human insulinoma. Biochem. Biophys. Res. Commun..

[64] Petersen H.V., Jorgensen M.C., Andersen F.G., Jensen J., Tove F.N., Jorgensen R., Madsen O.D., Serup P. (2000). Pax4 represses pancreatic glucagon gene expression. Mol. Cell Biol. Res. Commun..

[65] Wang Q., Elghazi L., Martin S., Martins I., Srinivasan R.S., Geng X., Sleeman M., Collombat P., Houghton J., Sosa-Pineda B. (2008). Ghrelin is a novel target of Pax4 in endocrine progenitors of the pancreas and duodenum. Dev. Dyn..

[66] Andersen F.G., Jensen J., Heller R.S., Petersen H.V., Larsson L.I., Madsen O.D., Serup P. (1999). Pax6 and Pdx1 form a functional complex on the rat somatostatin gene upstream enhancer. FEBS Lett..

[67] Aguayo-Mazzucato C., Koh A., El Khattabi I., Li W.C., Toschi E., Jermendy A., Juhl K., Mao K., Weir G.C., Sharma A. (2011). Mafa expression enhances glucose-responsive insulin secretion in neonatal rat beta cells. Diabetologia.

[68] Raum J.C., Gerrish K., Artner I., Henderson E., Guo M., Sussel L., Schisler J.C., Newgard C.B., Stein R. (2006). Foxa2, Nkx2.2, and PDX-1 regulate islet beta-cell-specific mafA expression through conserved sequences located between base pairs -8118 and -7750 upstream from the transcription start site. Mol. Cell. Biol..

[69] Raum J.C., Hunter C.S., Artner I., Henderson E., Guo M., Elghazi L., Sosa-Pineda B., Ogihara T., Mirmira R.G., Sussel L. (2010). Islet beta-cell-specific MafA transcription requires the 5'-flanking conserved region 3 control domain. Mol. Cell. Biol..

[70] Chou F.C., Shieh S.J., Sytwu H.K. (2009). Attenuation of Th1 response through galectin-9 and T-cell Ig mucin 3 interaction inhibits autoimmune diabetes in NOD mice. Eur. J. Immunol..

[71] Kanzaki M., Wada J., Sugiyama K., Nakatsuka A., Teshigawara S., Murakami K., Inoue K., Terami T., Katayama A., Eguchi J. (2012). Galectin-9 and T cell immunoglobulin mucin-3 pathway is a therapeutic target for type 1 diabetes. Endocrinology.

[72] Chou F.C., Kuo C.C., Wang Y.L., Lin M.H., Linju Yen B., Chang D.M., Sytwu H.K. (2013). Overexpression of galectin-9 in islets prolongs grafts survival via downregulation of Th1 responses. Cell Transpl..

[73] Li Y., Nagai H., Ohno T., Ohashi H., Murohara T., Saito H., Kinoshita T. (2006). Aberrant DNA demethylation in promoter region and aberrant expression of mRNA of PAX4 gene in hematologic malignancies. Leuk. Res..

[74] Lenoir O., Flosseau K., Ma F.X., Blondeau B., Mai A., Bassel-Duby R., Ravassard P., Olson E.N., Haumaitre C., Scharfmann R. (2011). Specific control of pancreatic endocrine beta- and delta-cell mass by class IIa histone deacetylases HDAC4, HDAC5, and HDAC9. Diabetes.

[75] Brink C., Chowdhury K., Gruss P. (2001). Pax4 regulatory elements mediate beta cell specific expression in the pancreas. Mech. Dev..

[76] Brink C., Gruss P. (2003). DNA sequence motifs conserved in endocrine promoters are essential for Pax4 expression. Dev. Dyn..

[77] Smith S.B., Watada H., Scheel D.W., Mrejen C., German M.S. (2000). Autoregulation and maturity onset diabetes of the young transcription factors control the human PAX4 promoter. J. Biol. Chem..

[78] Smith S.B., Gasa R., Watada H., Wang J., Griffen S.C., German M.S. (2003). Neurogenin3 and hepatic nuclear factor 1 cooperate in activating pancreatic expression of Pax4. J. Biol. Chem..

[79] Heremans Y., Van De Casteele M., in’t Veld P., Gradwohl G., Serup P., Madsen O., Pipeleers D., Heimberg H. (2002). Recapitulation of embryonic neuroendocrine differentiation in adult human pancreatic duct cells expressing neurogenin 3. J. Cell Biol..

[80] Kemp D.M., Lin J.C., Habener J.F. (2003). Regulation of Pax4 paired homeodomain gene by neuron-restrictive silencer factor. J. Biol. Chem..

[81] Martin D., Allagnat F., Chaffard G., Caille D., Fukuda M., Regazzi R., Abderrahmani A., Waeber G., Meda P., Maechler P. (2008). Functional significance of repressor element 1 silencing transcription factor (REST) target genes in pancreatic beta cells. Diabetologia.

[82] Martin D., Kim Y.H., Sever D., Mao C.A., Haefliger J.A., Grapin-Botton A. (2015). REST represses a subset of the pancreatic endocrine differentiation program. Dev. Biol..

[83] Mellado-Gil J.M., Fuente-Martin E., Lorenzo P.I., Bermudez-Silva F.J., Rojo-Martinez G., Romero-Zerbo S.Y., Campos-Caro A., Aguilar-Diosdado M., Gauthier B.R. (2016). The diabetes-link factor HMG20A maintains islet beta cell metabolic maturity. Diabetologia.

[84] Huotari M.A., Miettinen P.J., Palgi J., Koivisto T., Ustinov J., Harari D., Yarden Y., Otonkoski T. (2002). ErbB signaling regulates lineage determination of developing pancreatic islet cells in embryonic organ culture. Endocrinology.

[85] Holmstrom S., Van Antwerp M.E., Iniguez-Lluhi J.A. (2003). Direct and distinguishable inhibitory roles for SUMO isoforms in the control of transcriptional synergy. Proc. Natl. Acad. Sci. USA.

[86] Komatsu T., Mizusaki H., Mukai T., Ogawa H., Baba D., Shirakawa M., Hatakeyama S., Nakayama K.I., Yamamoto H., Kikuchi A. (2004). Small ubiquitin-like modifier 1 (SUMO-1) modification of the synergy control motif of Ad4 binding protein/steroidogenic factor 1 (Ad4BP/SF-1) regulates synergistic transcription between AD4BP/SF-1 and Sox9. Mol. Endocrinol..

[87] Chupreta S., Brevig H., Bai L., Merchant J.L., Iniguez-Lluhi J.A. (2007). Sumoylation-dependent control of homotypic and heterotypic synergy by the Kruppel-type zinc finger protein ZBP-89. J. Biol. Chem..

[88] Garcia-Dominguez M., Reyes J.C. (2009). SUMO association with repressor complexes, emerging routes for transcriptional control. Biochim. Biophys. Acta.

[89] Molvaersmyr A.K., Saether T., Gilfillan S., Lorenzo P.I., Kvaloy H., Matre V., Gabrielsen O.S. (2010). A SUMO-regulated activation function controls synergy of c-Myb through a repressor-activator switch leading to differential p300 recruitment. Nucleic Acids Res..

[90] Alm-Kristiansen A.H., Lorenzo P.I., Molvaersmyr A.K., Matre V., Ledsaak M., Saether T., Gabrielsen O.S. (2011). PIAS1 interacts with FLASH and enhances its co-activation of c-Myb. Mol. Cancer.

[91] Sireesh D., Bhakkiyalakshmi E., Ramkumar K.M., Rathinakumar S., Jennifer P.S., Rajaguru P., Paulmurugan R. (2014). Targeting SUMOylation cascade for diabetes management. Curr. Drug Targets.

[92] Aribi M. (2008). Candidate genes implicated in type 1 diabetes susceptibility. Curr. Diabetes Rev..

[93] Hajmrle C., Ferdaoussi M., Plummer G., Spigelman A.F., Lai K., Manning Fox J.E., MacDonald P.E. (2014). SUMOylation protects against IL-1beta-induced apoptosis in INS-1 832/13 cells and human islets. Am. J. Physiol. Endocrinol. Metab..

[94] Yang J., Zhang W., Jiang W., Sun X., Han Y., Ding M., Shi Y., Deng H. (2009). P21cip-overexpression in the mouse beta cells leads to the improved recovery from streptozotocin-induced diabetes. PLoS ONE.

[95] Bolhassani A., Jafarzade B.S., Mardani G. (2017). In vitro and in vivo delivery of therapeutic proteins using cell penetrating peptides. Peptides.

